# Using genomic data and machine learning to predict antibiotic resistance: A tutorial paper

**DOI:** 10.1371/journal.pcbi.1012579

**Published:** 2024-12-30

**Authors:** Faye Orcales, Lucy Moctezuma Tan, Meris Johnson-Hagler, John Matthew Suntay, Jameel Ali, Kristiene Recto, Phelan Glenn, Pleuni Pennings

**Affiliations:** 1 Department of Biology, San Francisco State University, San Francisco, California, United States of America; 2 University of California San Francisco, San Francisco, California, United States of America; 3 Department of Statistics, California State University East Bay, Hayward, California, United States of America; 4 David Geffen School of Medicine, University of California Los Angeles, Los Angeles, California, United States of America; SIB Swiss Institute of Bioinformatics, SWITZERLAND

## Abstract

Antibiotic resistance is a global public health concern. Bacteria have evolved resistance to most antibiotics, which means that for any given bacterial infection, the bacteria may be resistant to one or several antibiotics. It has been suggested that genomic sequencing and machine learning (ML) could make resistance testing more accurate and cost-effective. Given that ML is likely to become an ever more important tool in medicine, we believe that it is important for pre-health students and others in the life sciences to learn to use ML tools. This paper provides a step-by-step tutorial to train 4 different ML models (logistic regression, random forests, extreme gradient-boosted trees, and neural networks) to predict drug resistance for *Escherichia coli* isolates and to evaluate their performance using different metrics and cross-validation techniques. We also guide the user in how to load and prepare the data used for the ML models. The tutorial is accessible to beginners and does not require any software to be installed as it is based on Google Colab notebooks and provides a basic understanding of the different ML models. The tutorial can be used in undergraduate and graduate classes for students in Biology, Public Health, Computer Science, or related fields.

## Introduction

Penicillin was one of the first antibiotics that helped cure bacterial infections such as strep throat, gonorrhea, and meningitis [1]. Prior to the discovery of antibiotics, the average lifespan in the United States was merely 56 years, whereas in contemporary times, it has risen to approximately 80 years [2]. However, over the years, resistant strains of bacteria have evolved and become quite common, reducing the usefulness of antibiotics. As drug resistance has become more common, determining drug resistance phenotypes for pathogens is important for successful treatment of patients. Ideally, a doctor would know quickly which antibiotics they should prescribe to successfully treat their patient. Currently, this is usually done using phenotypic antimicrobial susceptibility tests that require significant time and resources in the laboratory. Genomic sequencing in combination with machine learning has been proposed as a way to accelerate this process [3].

Machine learning (ML) involves using large data sets to create models capable of making predictions [4]. Its applications in medicine are extensive from supporting roles such as handling medical health records, increasing the speed and accuracy of diagnosis of various diseases and in genetic engineering and genomics [5,6]. Studies have suggested that ML methods can identify tumors from medical images that may be overlooked by radiologists [7]. In recent years, many research groups have used ML to predict whether infections are caused by drug-resistant strains or not.

There is a lot of interest in using ML in combination with genomic data to predict antibiotic resistance [8–15]. Genomic data, including information on gene presence and absence, single-nucleotide polymorphisms (SNPs), indels (insertions and deletions), and k-mer frequencies, contain a lot of information that can be leveraged to predict phenotypes such as drug resistance. For example, Moradigaravand and colleagues used gene presence-absence information and population structure data to train various ML models to predict *Escherichia coli* resistance against 11 antibiotics [11]. Ren and colleagues led a similar study using SNP data and focused on 4 antibiotics [10]. Nsubuga and colleagues used SNP data to train ML models on data from England and then tested the models on data from several African countries [13]. In another study, Khaledi and colleagues used SNPs, gene presence-absence, and gene expression data to predict *Pseudomonas aeruginosa* resistance to 4 antimicrobial drugs [12]. A recent study by Hu and colleagues compared how a variety of ML methods were able to predict resistance phenotypes for 78 bacterial species-drug pairs [14].

The goal of this tutorial is to introduce the user to ML applied to genomic data to predict drug resistance, so they may potentially use it in their own future research. Note that we focus on the practical and intuitive side of ML rather than the bioinformatics part of a project (we start with an existing gene presence-absence table) or the mathematical details of machine learning. For an overview of useful bioinformatics tools, see [16].

With this paper, we aim to make ML more accessible for those who would like to learn it and who have an interest in biology or public health. The authors of this paper were a group of students (undergraduate, post-bacc, and master’s) at San Francisco State University that were excited to learn more about machine learning and its potential in antibiotic resistance research. At the start of this project, the authors were new to machine learning. As we developed the project, we gained experience in Python, genomic data, and creating ML models. This tutorial is a culmination of our efforts, learning journeys, and aims to serve those in a similar learning phase.

We used data and approaches from Moradigaravand and colleagues [11] with a few changes and simplifications. We chose this paper as the basis for this tutorial because it uses a large *E*. *coli* data set to predict resistance to 11 drugs and applies 4 common ML models. Their paper focused on predicting *E*. *coli* resistance in bacteremia patients from the United Kingdom. The models included in this tutorial are: extreme gradient-boosted tree, random forest, logistic regression, and neural network. For each of these models, we created a Google Colab Notebook and there are 2 additional notebooks for data preparation and to visualize the results. The benefit of coding in Google Colab Notebooks is that users can work in a browser with no need to install software on their own computer. Our goal was to create an easily understandable tutorial for anyone who wishes to learn more about ML and its applications in predicting antibiotic resistance.

## Data, machine learning models, cross-validation, and evaluation

### Data preparation

In this tutorial, we mostly use data and follow the approaches taken by Dr. Moradigaravand and colleagues [11] with some modifications for simplicity. While their approach is not the only way to use genomic data for ML purposes, it is a good place to start learning. We will work with a data set that was collected over many years in the UK [11]. For 1,936 patients who suffered from *E*. *coli* bloodstream infections, bacteria were isolated and tested against 12 antibiotics in the lab. Note that the Moradigaravand paper only reports results for 11 antibiotics, we will look at all 12 in the data set. An example of the phenotypic data can be seen in Table 1. Our goal here is to try and predict these antibiotic phenotypes using the genomic data from the same isolates.

**Table 1:** Example of antibiotic phenotypic data from the Moradigaravand dataset.

Strains	Ceftazidime	Cefotaxime	Ampicillin	Amoxicillin	Gentamicin	[…]	Ciprofloxacin
**Isolate 1**	S	S	S	S	S	…	S
**Isolate 2**	S	S	R	R	S	…	R
**Isolate 3**	S	S	S	R	S	…	S
**Isolate 4**	S	S	R	S	S	…	R
**Isolate 5**	S	S	R	R	S	…	S
**.**	.	.	.	.	.	.	.
**.**	.	.	.	.	.	.	.
**.**	.	.	.	.	.	.	.

**S:** Susceptible to the Antibiotic.

**R:** Resistant to the Antibiotic.

Bacterial genomes are usually sequenced using next-generation sequencing techniques. Starting from the raw sequencing reads, different bioinformatics tools are used for quality control, such as the removal of low quality sequences, to align reads to reference genomes and to create gene presence-absence tables and SNP tables. To build an ML model to predict drug resistance, we need to choose what information from each isolate to use for the model to learn from.

Moradigaravand and colleagues used 2 types of genomic data, but we will only be using one: the gene presence-absence table. We will not use the population structure data used by Moradigaravand. In addition to genomic data, Moradigaravand and colleagues used the year of isolation, which we will use here as well [11].

The gene presence-absence table summarizes for each bacterial isolate which genes are present or absent. Bacteria like *E*. *coli* can gain and lose genes through horizontal gene transfer, leading to different gene content in each isolate. Genes that confer resistance are among the ones being lost and gained, which is why a gene presence-absence table is expected to be relevant for predicting drug resistance, see Table 2.

**Table 2:** Example of gene presence-absence table.

Strains	Gene 1	Gene 2	Gene 3	Gene 4	Gene 5	[…]	Gene 17199
**Isolate 1**	1	0	1	0	1	…	S
**Isolate 2**	1	1	1	1	0	…	S
**Isolate 3**	0	0	1	1	0	…	S
.	.	.	.	.	.	.	.
.	.	.	.	.	.	.	.
.	.	.	.	.	.	.	.

**1:** Presence of gene in isolate.

**0:** Absence of gene in isolate.

While not used by Moradigaravand and colleagues [11], we decided to include the Multi-Locus Sequence Type (“MLST”) for each sample. Note however that we will not directly use MLST as a predictive feature to build the ML models. Instead, we will use it as an optional method to group the samples for our cross-validation step.

Other aspects of genomic data that are often used as features for machine learning but that we do not use here are SNP tables [10] and nucleotide or amino acid k-mer frequencies [17,18]. There are different reasons why researchers decide to use one type of genomic data or another. For example, to predict resistance that is caused by mutations, one may want to use SNP tables instead of a gene presence-absence table. This is particularly relevant for resistance to fluoroquinolones, such as ciprofloxacin, which is usually caused by mutations (SNPs) in *E*. *coli*. The benefit of k-mer frequencies on the other hand is that they can be calculated without aligning the sequences first.

The variables that are used as predictors in an ML model are usually referred to as features. We use 2 groups of features in this tutorial: gene presence-absence (G) and year of isolation (Y).

### Basic machine learning concepts

Common types of ML models include supervised learning, unsupervised learning, and reinforcement learning. In supervised learning, the model learns by recognizing patterns from a labeled data set. “Labeled” here means that the data set has an “answer key.” In our case, this means that we work with isolates for which we already know whether they are resistant or susceptible to different drugs thanks to tests done in a laboratory (see Table 1). These labels are used to train the ML model, and also, afterwards to test how well the model works. Before training an ML model, the entire data set is split into a **training data set** and a **testing data set**. Usually, around 70% to 80% of the data are used as the training data set and the rest is used for testing. In our notebooks, we used 80% training data and 20% testing. The training data set is used for the model to learn the patterns associated with resistance (or susceptibility). After that, the testing data set is used to determine how well the model can predict the label.

### Features and labels

Features in ML can come in different formats, depending on the type of data used, such as pixel arrangements from an image or a set of words in a sentence, but it ultimately ends up coded numerically. For this tutorial, the features are already organized as tabular data, where each row is an observation (here a bacterial isolate) and each column is a feature. Each gene becomes a feature that is coded with 1 or 0, and the year of isolation is also a numeric feature that ranges from 1970 to 2017. The training data contains **features** that the model will attempt to learn patterns from and the **labels** it learns to predict. Here, the features are a list of genes for each isolate with indicators of whether a particular gene is present (1) or absent (0), and the year the isolate was collected. For each drug, we create a separate model where the label we are trying to predict is whether the isolate is resistant or susceptible to that drug. Isolates listed as “intermediate” are considered resistant.

After the ML model is trained using the **training data** (features and labels), the features of the **testing data** will be used for the ML model to make label predictions. The predictions will then be compared with the known labels for the testing data set. We can then determine how often the model gets it right and how often it gets it wrong.

### Four supervised classification models

Within the Google Colab notebooks, the user will be guided on how to implement Logistic Regression, Random Forest, Extreme Gradient-Boosted Trees, and Neural Networks models to predict antibiotic resistance in *E*. *coli*. Each model will be briefly explained below, and further explanations are provided in the notebooks. For each of the models, we will guide the user in assessing the quality of the predictions.

**Fig 1:**
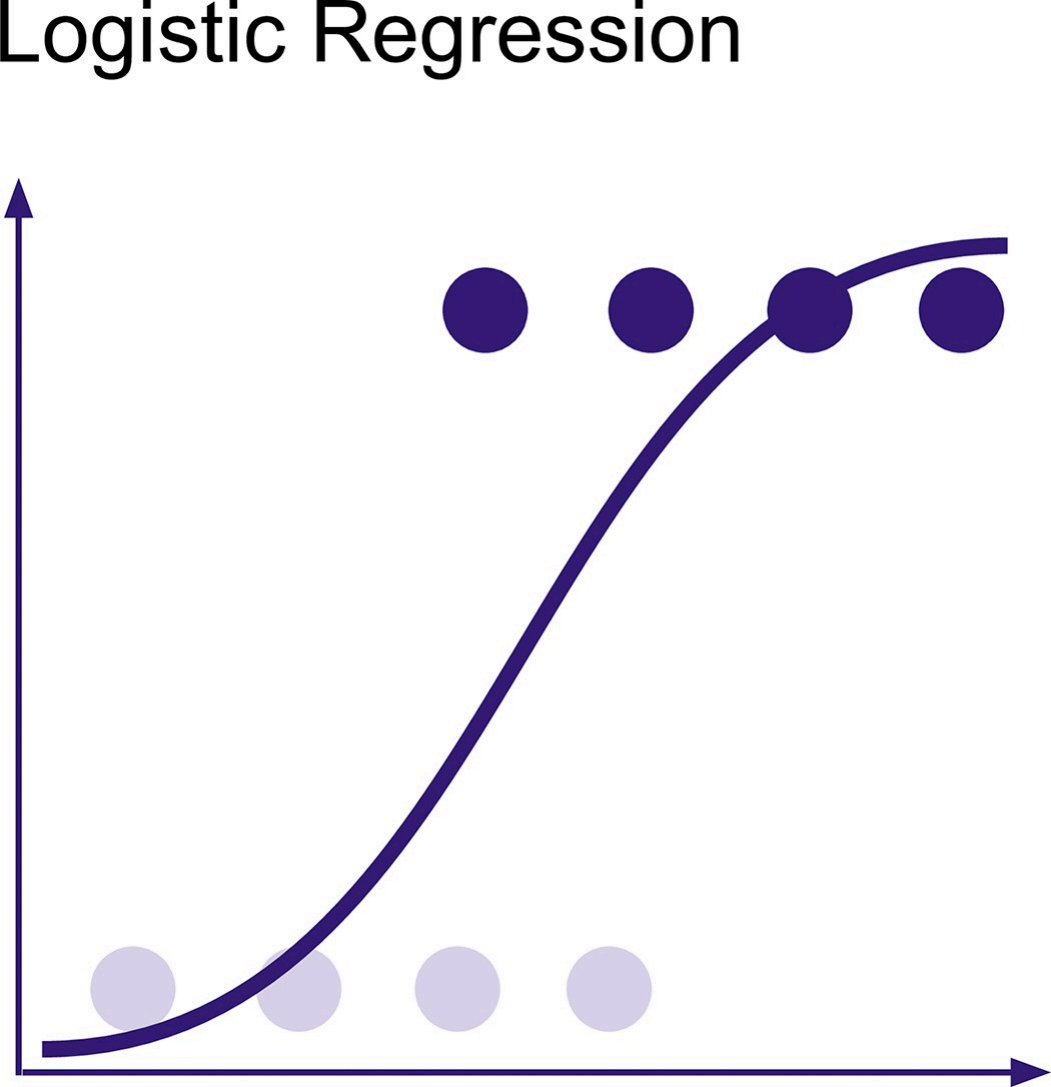
Logistic Regression. A sigmoid function that is representative of a logistic regression model.

**Logistic Regression** is a classification model that predicts the probability for a binary outcome (2 classes), such as susceptible and resistant (see Fig 1). Logistic regression models have specific probability thresholds that the model uses to decide what class a particular observation belongs to: the susceptible or resistant class. The default threshold for logistic regression is 0.5. Above this value, the model predicts one class; below, it predicts the other class [19].

In the trained model, each gene in the gene presence-absence table will have a coefficient associated with it in the logistic regression model. If that coefficient is positive, it means that presence of the gene increases the probability that the model predicts that the sample is resistant. If the coefficient is negative, presence of the gene reduces the probability the model predicts resistance.

**Fig 2:**
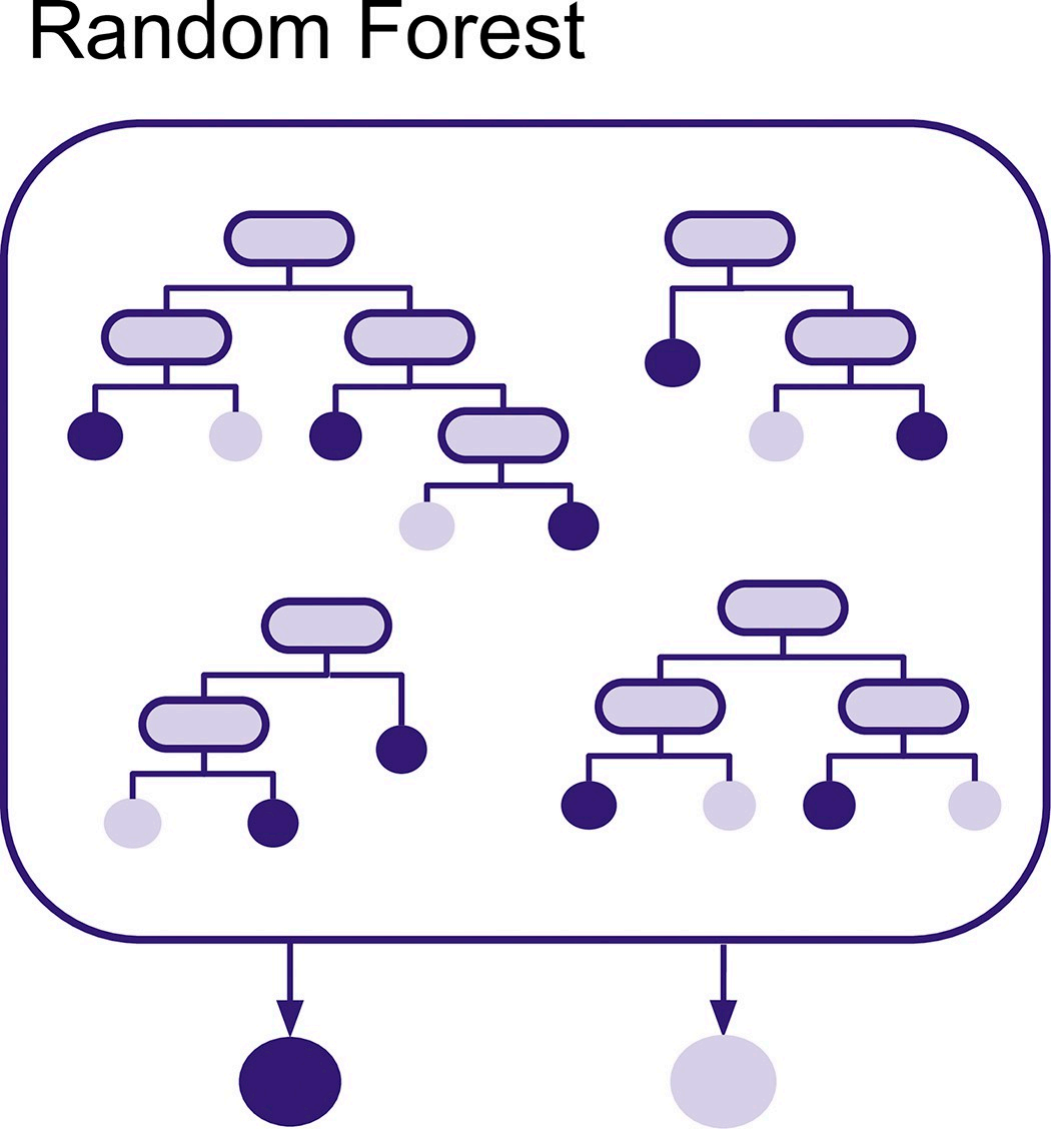
Random Forest. Multiple decision trees that are the basis of a random forest model.

A **Random Forest** is an ensemble method and as such combines outputs from multiple simpler models, in this case from a collection of decision trees (see Fig 2). Each decision tree independently classifies samples using a series of yes/no questions, and the final decision for each observation is based on the most common prediction among the trees [20]. Yes/no questions used for decision trees are based on the features, for example, whether an isolate carries a specific gene. When building a tree, an algorithm tries to find the best yes/no questions to group the observations into groups that are mostly resistant or mostly susceptible. For example, for predicting amoxicillin resistance, the first split may be based on whether or not the isolates carry a *Bla* gene or not. Those who carry the *Bla* gene are often resistant, whereas those who do not carry that gene are most often susceptible. Subsequent splits of the tree will use other features to keep classifying observations until the entire tree is built.

The randomness of the Random Forest comes from the fact that each tree is made using random subsets of the data. First, each tree in the Random Forest is created using a random subset of observations via bootstrapping (a selection of data points chosen at random with replacement). Second, each node for each of the trees is created using a subset (without replacement) of the features.

Based on the default hyperparameters as of version 1.4.2 of the skit-learn version release, a Random Forest will consist of 100 trees. If a data set has 900 features and 200 observations, then each decision tree will be based on a bootstrap sample (that is, sampling with replacement) of 200 observations from the original set of observations. For each node of each tree, a subset of features of size sqrt(900) will be searched for the best split for that node. In other words, for each node, 30 features are chosen at random and the best of those 30 is used for that node. This process is repeated for each node for each decision tree in the random forest. After the Random Forest is created, it can be used to make predictions for the test data set. To do that, each observation is classified by all decision trees in the random forest. If an observation is classified as resistant by 80 trees and classified as susceptible by 20 trees, then the final prediction of the Random Forest will be resistant.

**Fig 3:**
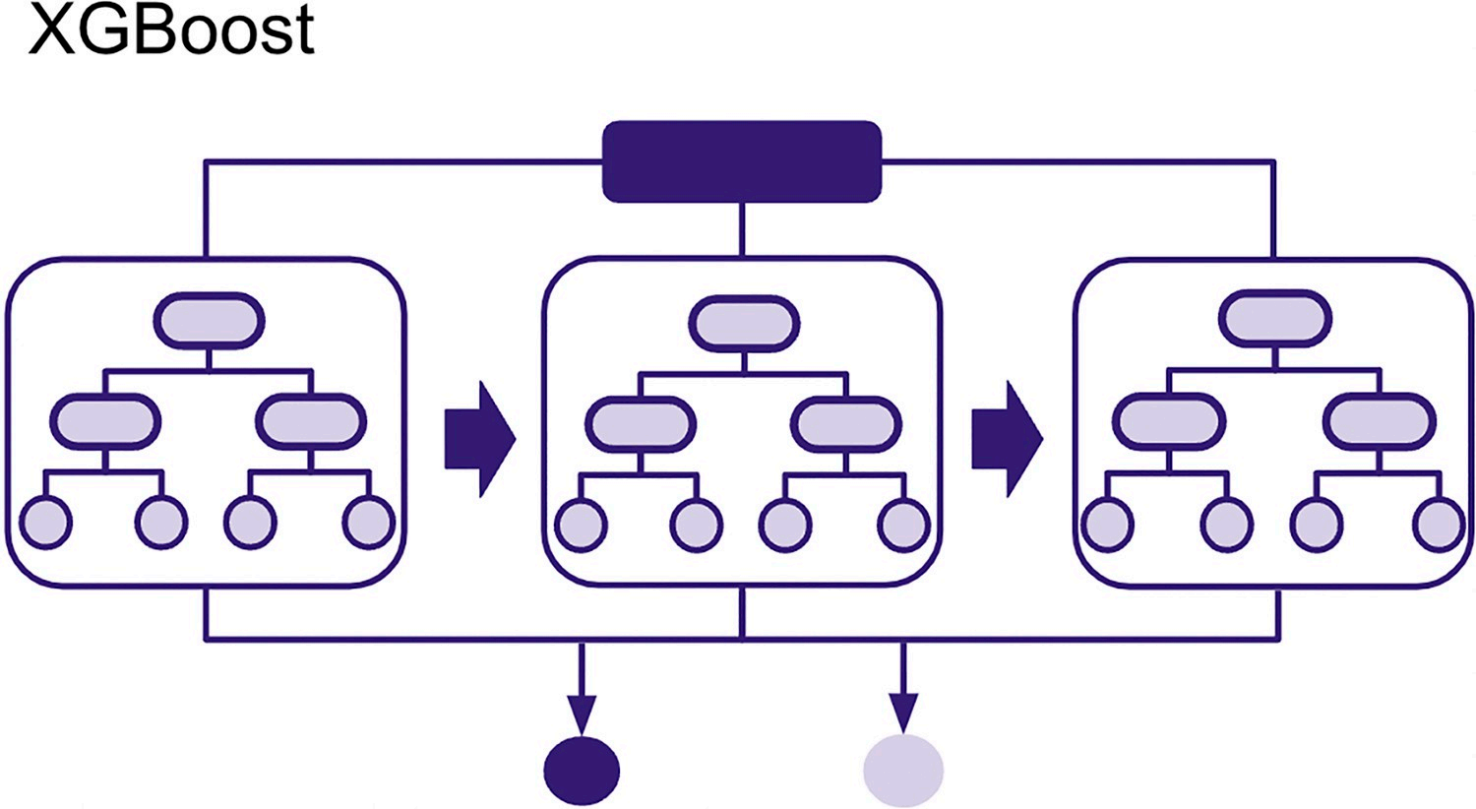
Extreme Gradient-Boosted Tree. Decision trees created sequentially in Extreme Gradient-Boosted Tree (XGBoost).

An **Extreme Gradient-Boosted Tree** is another ensemble method that can be used for regression and classification tasks (see Fig 3). Like Random Forests, it also uses Decision Trees. Each tree also uses a random subset of observations, but this time without replacement. This means that the unique observations picked by a decision tree will not be duplicated. One of the main differences between Random Forests and Extreme Gradient-Boosted Trees is that the individual trees here do not make predictions on the target label, instead they attempt to make predictions on pseudo-residuals also known as leaf output values. These output values are named pseudo-residuals because they are mathematically derived from actual residuals (i.e., the difference between the actual or true probability of classes (0 or 1) in our training data and the ones predicted for each iteration of a decision tree) [21] using a mathematical function that gets updated through several iterations, each iteration depending on the previous tree’s leaf outputs. Through this method, the trees are created sequentially, with the next tree attempting to improve the prior one by reducing these pseudo-residuals. At the end of the training, all tree leaf outputs created are used in the final prediction.

**Fig 4:**
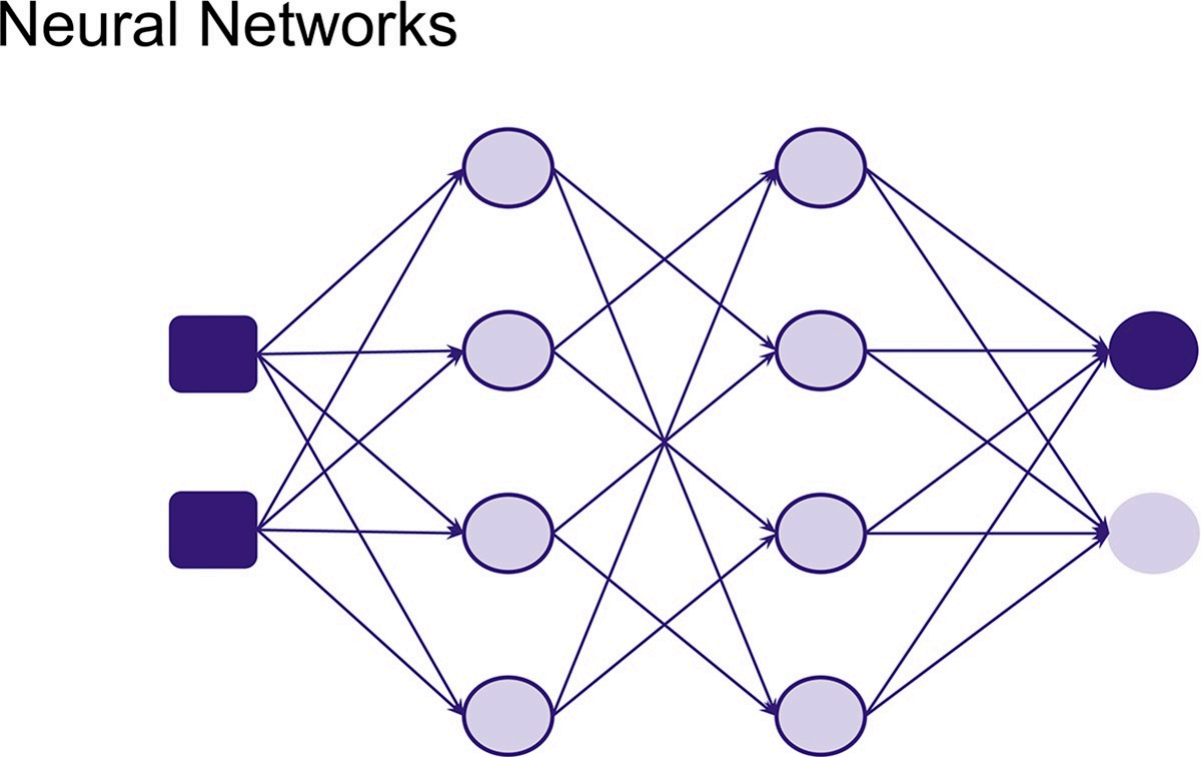
Neural Networks composed of different nodes inspired by neurons.

A **Neural Network** is a method where the model learns to make predictions through a trial-and-error approach as well as an iterative analysis of training samples (see Fig 4). They are loosely inspired by how biological neurons are connected and signal each other. This model serves as the foundation for different types of Neural Network architectures. Different kinds of data can be used to train neural networks, such as images and sounds, etc. Depending on the type of data and task, a specific Neural Network architecture can be used. Although usually requiring more data and computational power, more complex neural networks have been shown to be able to predict antimicrobial resistance taking into account gene interactions [22]. But in this tutorial, we will keep it simple and learn about how to construct a neural network from scratch and train it with simple tabular data.

### Cross validation

**Fig 5:**
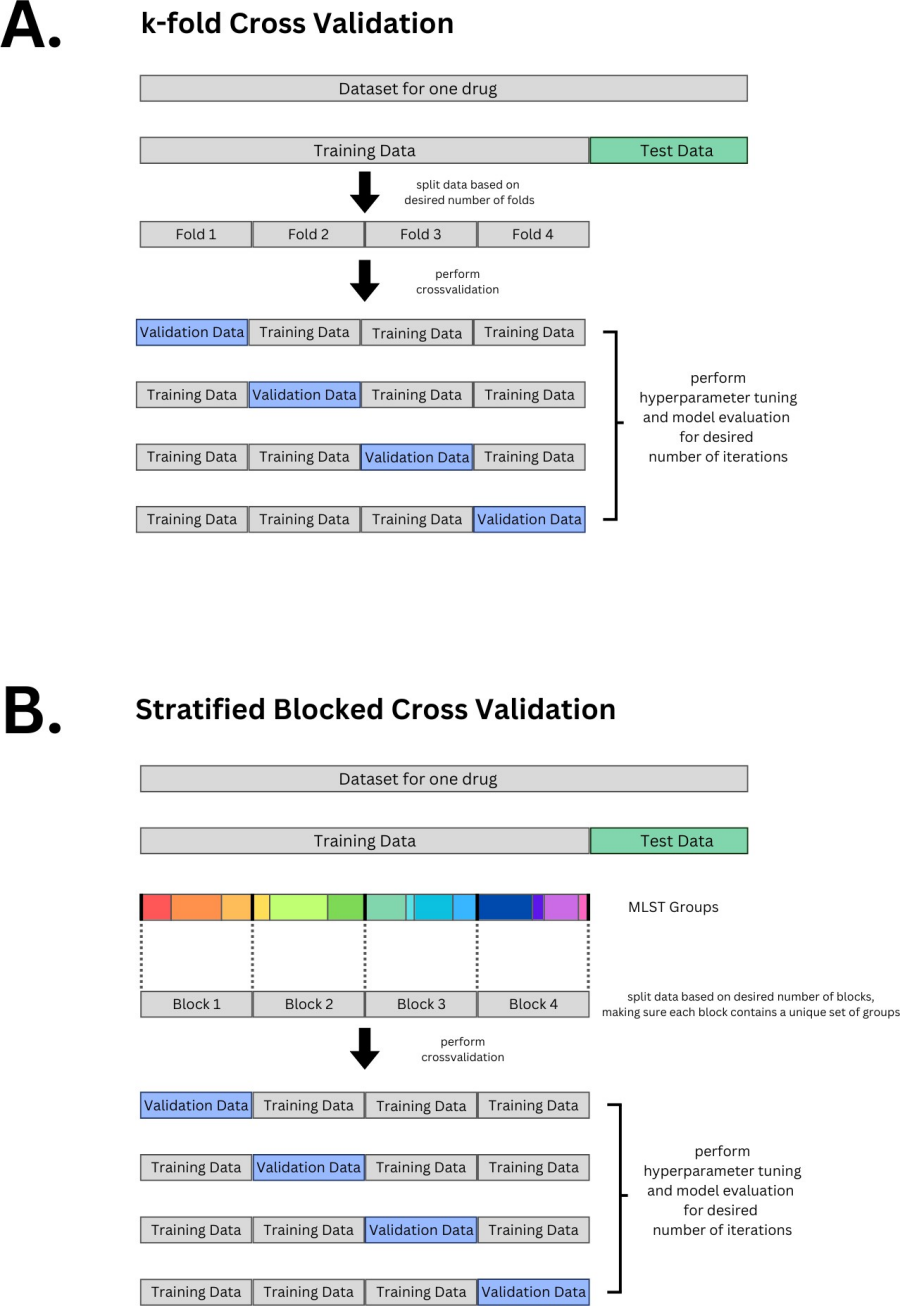
Cross Validation. **(A)** k-fold cross validation (here shown is 4-fold). **(B)** Stratified blocked cross validation. Code is provided for both options: regular random k-fold and for stratified blocked cross validation based on MLST. For both options, the initial training and testing data split is stratified based on labels (R, S).

Cross validation is a commonly used method in machine learning. It has several purposes such as making sure the model generalizes (is able to predict well on data it has never seen before). Here, we will use cross validation to do hyperparameter tuning. Cross validation works by taking out a subset of the training data to use as a validation set, while the remaining subsets are used for training the model. This process is then repeated with different validation sets. This will allow us to monitor the model’s performance on a diverse set of validation subsets. There are different strategies for choosing the subsets for cross validation. We will discuss 2 methods: regular k-fold cross validation and stratified blocked cross validation. The k-fold cross validation method works by splitting your data into equal groups or “folds.” For example, if we use 4 folds the data is divided equally into 4 subsets. One of them is held out and used as the validation data set while the rest are all used for training. This is repeated until each unique fold has been used as the validation data set once (Fig 5A). It is up to the user how many folds you decide to use. The higher the number of folds you set, the longer the computational time.

The stratified blocked cross validation method also splits the data into folds; however, it differs in how each fold is assigned a validation and training chunk. “Stratified” here means that the different folds try to maintain similar label proportions, for example, if the training folds contain about 70% Susceptible and 30% Resistant isolates, then the validation fold tries to maintain this proportion as well. “Blocked” here means that isolates are grouped into different MLST groups, where the MLST groups present training folds are not present in the validation folds. This is one of the methods used to ensure generalizability when training our model (Fig 5B).

### Evaluating model performance

**Fig 6:**
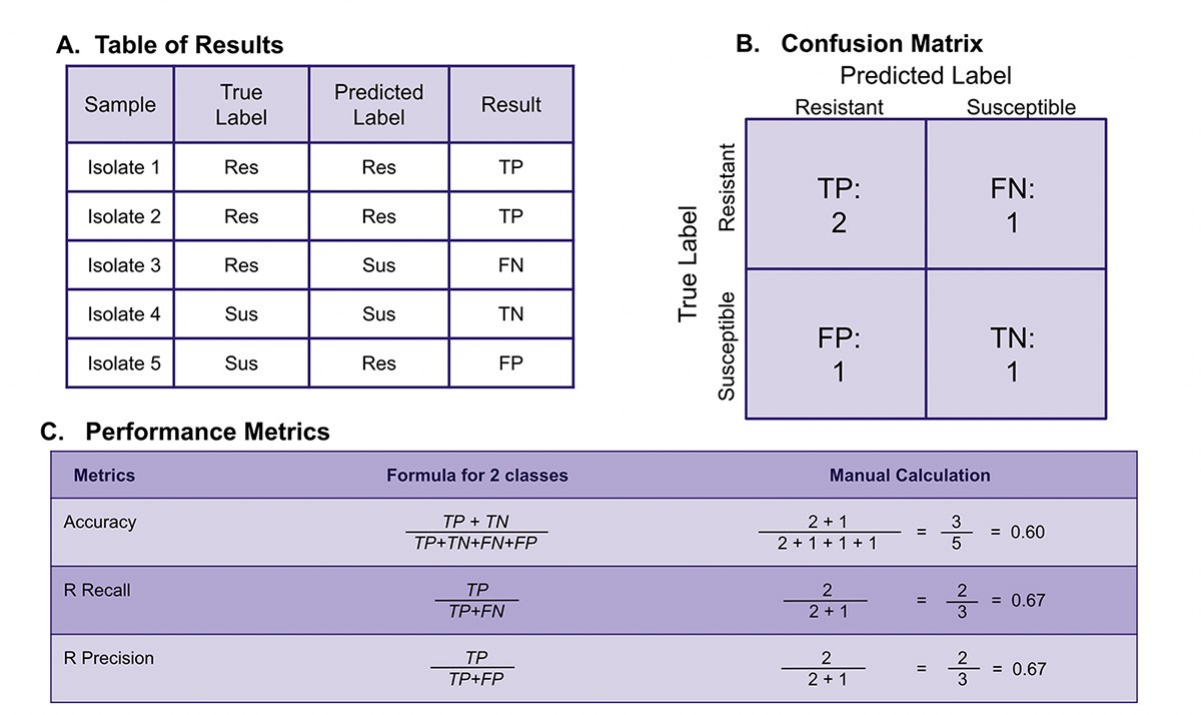
Example of how confusion matrix results are used to calculate evaluation metrics. **(A)** Determination of true positive (TP), true negative (TN), false positive (FP), and false negative (FN) labels can be seen for toy examples. **(B)** A confusion matrix based on the toy model from **(A)** showing total numbers of TP, TN, FP, and FN. **(C)** Numbers from the confusion matrix are used to calculate various evaluation metrics.

After training the model (and during cross validation), it is important to evaluate the model’s performance. There are many evaluation metrics, but we will use some of the most common ones used for classification tasks which can be derived from a confusion matrix: accuracy, recall, and precision. These metrics are used to quantify how well the model performs. In Fig 6, we illustrate how different metrics are calculated from a confusion matrix using a toy example.

The most common metrics for classification models are based on the amount of true positive (TP), true negative (TN), false positive (FP), and false negative (FN) predictions. A true positive or true negative outcome is when a model predicts the resistant or susceptible class (respectively) correctly from the test data we gave the model. Conversely, a false positive or false negative outcome is when a model predicts the resistant class incorrectly (e.g., classifying the strain as susceptible when it is actually resistant results in a false negative).

Three of the most common evaluation metrics that can be calculated from these true/false positive/negative outcomes are accuracy, recall, and precision. **Accuracy** is the number of correct classifications over the total number of predictions made. **Recall** is the number of correct classifications made for a particular class over all samples in that class. In our case, resistance recall refers to the fraction of resistant isolates that were correctly classified as resistant, out of all the true resistant isolates. **Precision** is the number of correct classifications made for a particular class over the total number of samples that are predicted to be in that class—in our case, this means the fraction of correctly labeled resistant isolates divided by all isolates that were predicted to be resistant. The formulas used to calculate these metrics are provided in Fig 6.

Generally, a model that consistently scores close to 1 for accuracy, recall and precision is considered to be well-performing. For the case of resistance, it may be most important to have high recall for resistance (making sure we “catch” all of the resistant strains)—even if the other evaluation metrics are not that high. A resistance recall score close to 1 means that the model was able to correctly identify many resistant strains (high true positive cases), while also having low instances of predicting a strain was susceptible when it was actually resistant (low false negative cases). If most isolates are susceptible, it is possible to have a high accuracy even if the model does not correctly predict any of the resistant strains. For example, if 95% of the isolates in the data set are susceptible, and a model predicts that all isolates are susceptible, then the accuracy would be 95%, because the model gets it right for 95% of the cases; 95% accuracy sounds great, but the recall for resistance would be 0%, which shows that the model is not useful at all. Finally, we would like to mention another common metric for how well an ML model performs is the F1 score, which is calculated as the harmonic mean of precision and recall.

You will also observe evaluation metrics during cross validation/hyperparameter tuning. The metrics during cross validation, which are acquired from the specific combination of hyperparameters tested in each validation fold, allows us to peek at the performance during the training phase; these should give us an initial clue to how the models will actually perform when dealing with test data. In our tutorial, we use the F1 macro score as a metric we want to optimize. The F1 macro score is essentially the average F1 score for each of the classes (R & S). Optimizing this value tries to increase all the other metrics: recall, accuracy, and precision, without giving higher priority to any particular class or metric.

## Overview of the tutorials

The typical ML analysis pipeline involves preparing data, using that data to train an ML model and testing how well the model performs. The tutorials provided will go through each step of this pipeline to create models that can predict drug resistance based on genomic data. The tutorials are written in Python, as this is the most commonly used language in machine learning. In order to make the tutorials easily accessible, we have provided them in Google Colab format as this allows for the notebooks to be used in a browser, without having to set up a coding environment on a computer. To go through all the steps of the tutorial, one needs to have a Google account. Furthermore, all of the libraries and tools (listed in Table 3) used in the tutorials are free to access.

The tutorial is made up of 6 notebooks.

1. **Loading and Preparing data for ML models**
2. **Logistic Regression**
3. **Random Forest**
4. **Gradient-Boosted Trees**
5. **Neural Network**
6. **Visualization to compare results from all 4 ML models**

All notebooks, the data, and csv files with results can be found in a GitHub repository.

**Table 3:** Python Libraries used in tutorials.

Python Libraries	Documentation links
**pandas**	https://pandas.pydata.org/docs/user_guide/index.html
**numpy**	https://numpy.org/doc/stable/user/index.html#user
**random**	https://docs.python.org/3/library/random.html
**functools**	https://docs.python.org/3/library/functools.html
**os**	https://docs.python.org/3/library/os.html
**sklearn**	https://scikit-learn.org/stable/user_guide.html
**matplotlib**	https://matplotlib.org/stable/users/index
**seaborn**	https://seaborn.pydata.org/tutorial.html
**graphviz**	https://graphviz.org/documentation/
**xgboost**	https://xgboost.readthedocs.io/en/stable/python/index.html
**tensorflow**	https://www.tensorflow.org/guide
**keras**	https://keras.io/guides/

The notebooks include all code that is needed plus extensive explanations of the code. The first notebook has the code for loading and preparing the data (Data Preparation). The objective of this notebook is to understand some basic background information about antimicrobial resistance and genomic data for those who are new to the subject, then we combine the data sources into a single csv file, which will be saved to the user’s Google Drive folder and which will be used in the following notebooks to train the ML models (second to fifth).

In each of the next 4 notebooks, we will go through a basic pipeline for ML analysis. We first load the csv with combined features, next we split our data into training and testing, and create different feature combinations (Y (Year) only, G (gene presence-absence) only or G and Y both). Then, we create and train a model. Throughout, some of the technical details are explained about the specific model, for example, understanding how random forests make a classification, how to get feature importance from an XGBoost model or how layers are constructed for a neural network, etc. Afterwards, hyperparameter tuning and cross validation are performed. In order to keep the tutorial simple, we restricted ourselves to tuning only 1 to 2 parameters; however, the code is laid out in a way that makes it easy to try different hyperparameters and values through the use of python dictionaries. The tutorials also include the option to perform either random k-fold cross validation or stratified blocked cross validation.

Once the model has been trained and hyperparameter tuned, its performance is evaluated by having it predict the labels of the test data set. To do this, we use a custom function which computes evaluation metrics (accuracy, recall and precision scores for each label class) for a total of 36 models (12 antibiotics × 3 feature combinations). The same workflow is used in all the ML model notebooks: Logistic Regression (Logistic Regression), Random Forest (Random Forest), Extreme Gradient-Boosted Tree (Extreme Gradient-Boosted Tree), and Neural Network (Neural Network).

**Fig 7:**
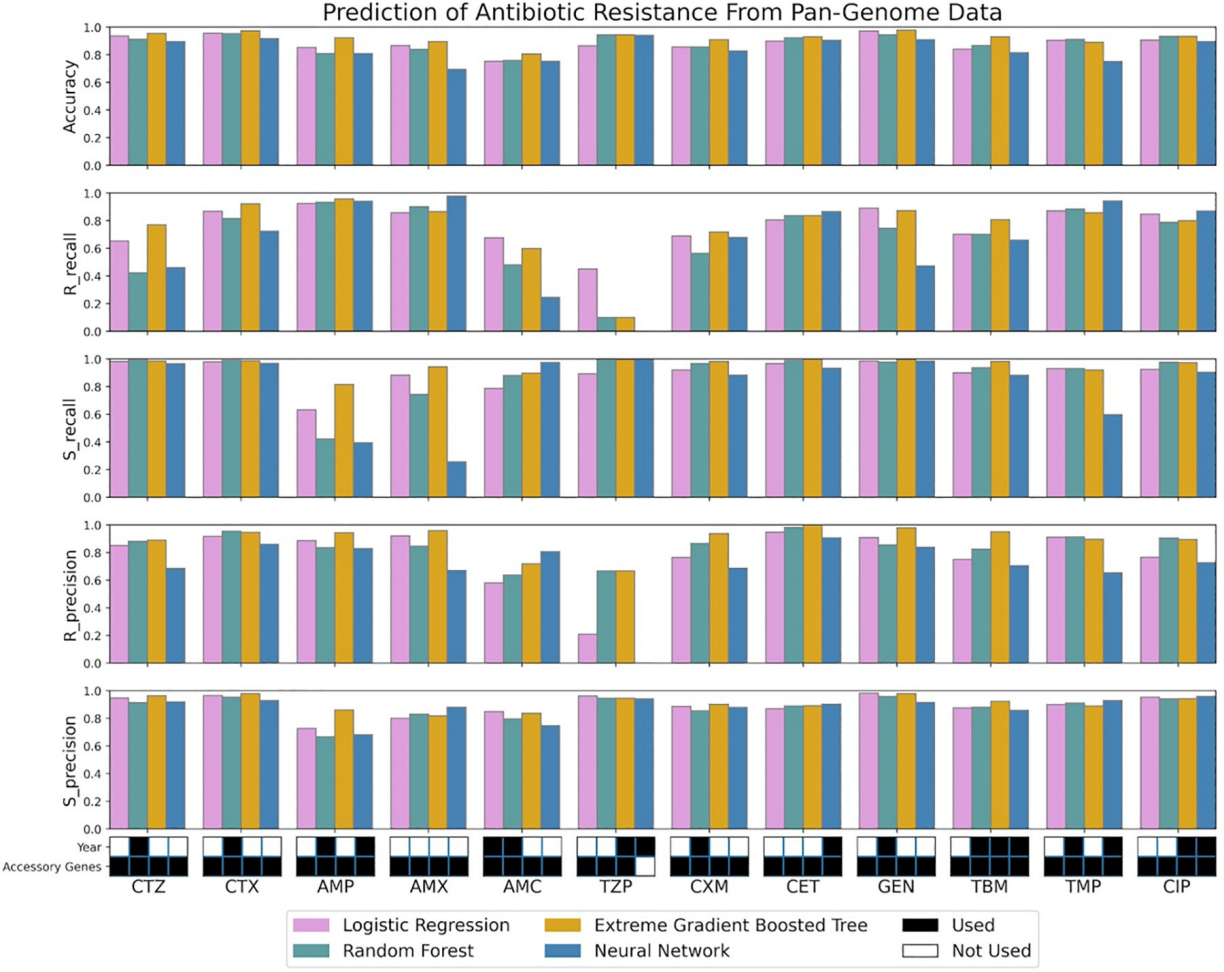
Final data visualization result from the last tutorial notebook. The figure shows various bar graph subplots, with each one showing results for an individual evaluation metric. Only the highest-performing combinations of features for each metric are shown. The color key refers to the machine learning models (pink for logistic regression, green for random forest, yellow for extreme gradient-boosted trees, and blue for neural network). The black and white grid on the very bottom indicates which features were used to result in the highest performing model that predicted each antibiotic: ceftazidime (CTZ), cefotaxime (CTX), ampicillin (AMP), amoxicillin (AMX), amoxicillin-clavulanate (AMC), piperacillin-tazobactam (TZP), cefuroxime (CXM), cephalothin (CET), gentamicin (GEN), tobramycin (TBM), trimethoprim (TMP), ciprofloxacin (CIP).

Finally in the last notebook, we focus on comparing the different ML models and visualizing the best yielding results (Results Visualization). The final part of our tutorial shows how to use the Matplotlib and Seaborn libraries to create bar graphs for the best scores obtained for each drug in all 4 models (see Fig 7). Note that Fig 7 is based solely on the results using random k-fold cross validation.

The results in Fig 7 show that while accuracy is quite high for most drugs and most ML models, R recall is very variable. For example, our models do a good job identifying ampicillin resistant strains (R recall for ampicillin is around 90%), but not at all for TZP (Tazobactam, recall around 10% or lower for 3 out of 4 ML models). There could be many reasons for this low performance including, but not limited to: a very unbalanced data set, not enough hyperparameter tuning, or not including the right data. For TZP, it is certainly true that the data set is unbalanced with only 101 resistant samples and 1,575 susceptible samples. This makes it hard for a model to learn to recognize the resistant samples.

When we compare the models, the extreme gradient-boosted tree model has the highest accuracy and recall for many of the drugs. Fig 7 also shows at the bottom of the figure which features are included in the best model. We see that gene presence-absence (G) is always included, while year (Y) is only included about half of the time. There is 1 exception: the neural network model for TZP only includes year, but not the gene presence-absence data—however, this model performs very badly and has 0 recall for resistant samples.

## Discussion

The objective of this manuscript and the tutorial was to create a user-friendly guide to the application of ML for predicting antibiotic resistance. We designed 6 notebooks to introduce concepts and methods that are needed in such an analysis, including data wrangling, machine learning classification models, and data visualization. Preprocessed data was provided in the first notebook as a starting point and context for our tutorial. All notebooks contain a step-by-step explanation detailing how we developed an ML pipeline to analyze *E*. *coli* genomic data in order to predict resistance. In order for new users to better develop an understanding of ML methods and applications, modular code chunks were implemented and annotated in all notebooks. All notebooks for this tutorial are open access and available for execution in Google Colab.

While our main goal was to demystify machine learning for those with a background in biology, we hope that users of our materials will also appreciate that there are many different ways to train ML models to predict drug resistance and many choices that a researcher needs to make [3,23,24].

For most ML models, numerical data is required, which requires the transformation of the raw sequencing data or other available data. In this tutorial, we used a gene presence-absence table (a common approach) and the year of isolation (an uncommon approach). Other common options include SNP tables and k-mer frequencies. For example, Nguyen and colleagues’ paper focuses on k-mer frequencies for a small set of core genes [25]. Next, the type of model should be chosen. Here, we used 4 types of models (logistic regression, random forest, extreme gradient-boosted trees, and neural network). Other options we did not use include support vector machines and K-Nearest Neighbors (KNN).

An important issue in machine learning for any biological samples is how to deal with relatedness. For example, it could be that some of the *E*. *coli* isolates in the data set are very closely related to each other (maybe they were part of a hospital outbreak). If some of these isolates are in the training data and some in the test data set, then it could be that the ML model learns to recognize the strain, and can therefore predict its resistance phenotype. This could be a problem if we wanted to use the model later on with other data that contains different strains [13]. In other words, such a model may not generalize well. One way to deal with this is to make sure that related strains are either all in the training data or all in the test data. A common way to implement this is through the use of a blocked cross-validation scheme. Because related isolates (same MLST group) used in training are evaluated against a different MLST group in the validation folds. If we observe good metrics in that case, it means that the model is likely generalizable to other MLST groups. A blocked cross-validation method has been used in other papers such as [14], where it is compared against the regular random k-fold cross-validation. The paper finds that when blocking is used, cross validation scores tend to be lower than using random cross validation, lending support to the idea that taking population structure into account is important when applying ML using genomic data. Following this example, our tutorial provides both options. Our tutorial also deals with class imbalance. Because for most drugs there are more susceptible strains than resistant ones, our initial train-test split was based on label (R/S) and our block cross validation was also stratified by label as well.

An important lesson to learn for anyone starting with machine learning is that the patterns that are picked up by the models are not necessarily causal. For example, in the random forest notebook, when we looked at the feature importance, the most important feature in the random forest model was a gene named *wbuC*. As far as we are aware, this gene does not cause resistance. However, it appears to be a neighbor of *mcr9*.*1* in some plasmids and this is a known resistance gene [26].

It is important to consider different performance metrics. We have focused here on accuracy, recall, and precision. We believe that recall for resistance may often be the most important metric, especially if resistance is quite rare. Resistance recall tells us what fraction of resistant isolates is detected by the model. When resistance is rare, it is quite common that accuracy is high, but recall for resistance is low. In such a case, the model predicts “susceptible” in most cases and is right in most cases. Yet, the few resistant isolates are not detected, making the model useless in clinical practice. We experienced this issue with our TZP data, where we saw relatively low resistant recall scores despite having relatively high accuracy and susceptibility recall scores (Fig 7). A common metric which we did not include in the tutorial is the F1 score. F1 is calculated as is the harmonic mean of precision and recall. While useful, we believe that for most humans, recall is much easier to understand.

After working through the tutorial, we hope that users will be encouraged to continue learning. One could take the existing code and use hyperparameter tuning to improve the performance of the model. For example, one may want to find the best tree depth for the ensemble models in our tutorial, or the ideal number of layers for the neural network model. Within each model, there are parameters that can be tweaked to improve the models we have provided. Information on the parameters available for each model can be found in the documentation online (see links in Table 3). The documentation also specifies the default value for each parameter and the range of possible values that can be used.

In addition to improving the models for the data provided, users may be interested to use other models on the same data sets or to use the same models on other data sets. There are many bacteria across the world that are developing antibiotic resistance that need to be analyzed such as *P*. *aeruginosa*, *M*. *Tuberculosis*, *S*. *aureus*, and many others.

Our tutorial only scratches the surface of how ML models can be used to predict resistance phenotypes in bacteria. We made this tutorial to make it easier for others to start learning about machine learning and how it is applied to genomic data sets to predict resistance. We hope that our tutorial will help more people learn these skills and ultimately lead to better outcomes for patients.
